# Risks Seem Low While Climbing High: Shift in Risk Perception and Error Rates in the Course of Indoor Climbing Activities

**DOI:** 10.3389/fpsyg.2018.02383

**Published:** 2018-12-17

**Authors:** Martina Raue, Ronnie Kolodziej, Eva Lermer, Bernhard Streicher

**Affiliations:** ^1^AgeLab, Massachusetts Institute of Technology, Cambridge, MA, United States; ^2^Department of Psychology, Ludwig-Maximilians-Universität München, Munich, Germany; ^3^Department of Experimental Psychology, University of Regensburg, Regensburg, Germany; ^4^FOM University of Applied Sciences for Economics and Management, Munich, Germany; ^5^Department of Psychology and Medical Sciences, UMIT-Private University for Health Sciences, Medical Informatics and Technology, Hall in Tyrol, Austria

**Keywords:** risk perception, physical activity, climbing, mountaineering, embodiment, extreme sports

## Abstract

Little is known about people’s risk perception *while* participating in potentially harmful activities. In a field study conducted in a climbing gym, we investigated how the climbing activity influences climbers’ risk perception and error rates. Based on research on embodiment, we argue that the perception of risks may differ between being in an active state during climbing and being in an inactive state before or after climbing. In addition to subjective risk perception, error rates were observed as an objective measure of behavior that increase the risk for accidents. Experience was included as a potential moderator because indoor climbing is also accessible to inexperienced people. Drawing on the affect heuristic, we hypothesized that experienced climbers are guided by their intuitions, which makes them less susceptible for influences of their physical state when judging climbing risks. Less experienced climbers need to rely more on deliberative thinking and their judgments may be more distorted by the effects of being in an active state. Climbers were asked to judge climbing risks at five points in time (twice before, twice during and once after climbing in a gym). In addition, the amount and type of climbing errors was observed at two points in time during the activity. We recruited 57 participants (32% female) in a large climbing gym in Germany, who were between 18 and 57 years of age. Results show that participants’ perception of climbing risks generally decreased during the activity phase as opposed to the pre-activity phase, while error rates increased. Higher experience was associated with lower risk perception, but also more errors. Further, experience may weaken the influence of physical activity on risk perception. In higher risk sports people have to make important decisions while being active. Our results suggest that especially climbers need to be aware that being physically active can distort their risk perception.

## Introduction

Leisure activities that are potentially harmful, such as climbing, backcountry skiing, mountain biking or white-water rafting, have become increasingly popular in recent years (Pain and Pain, 2005; Brymer and Schweitzer, 2017). Formerly practiced by few people, these activities are now practiced by many worldwide, and have, thus, become a societal phenomenon. From a social psychological perspective, however, little is known about people’s risk perception *while* participating in these activities. This was investigated at the example of an indoor climbing activity in the following study.

Indoor climbing has become very popular in recent years and climbing gyms can be found in almost every larger city in central Europe and the United States. Those gyms consist of high walls with artificial holds and designated routes of various levels of difficulty. A partner belays the climber with a rope attached to a harness. Indoor climbing incorporates various safety measures and severe accidents are rare. However, they do happen, often due to human error (Neuhof et al., 2011; Schöffl et al., 2013, 2015). A recent exploratory study on climbing gym culture suggests that low levels of perceived social control by fellow climbers and little observance by staff can increase the risk for accidents (Schwiersch et al., 2015). A climber may perceive the potential risks as being very low and therefore not take safety recommendations seriously. Accidents in higher risk sports can result from deliberate risk-taking or a lack of precautionary measures (Woodman et al., 2013). A lack of precautionary measures may include skipping the recommended partner check (each climber checks the partner’s harness, the knots and the belay-system) or a lack of concentration while belaying. In addition, climbers may behave riskily *while* climbing on the wall because they may overestimate their skills or lack concentration, which can result in severe errors. In this article, we argue that it may be possible that being in a state of physical activity during climbing leads to an underestimation of risks. Thus, the perception of risks may differ between being in an active state during climbing and being in an inactive state before or after climbing.

Higher risk sports, such as rock climbing or backcountry skiing, include potential threats, but the actual probability of a certain individual to experience injury or harm in the given situation is unknown. Because the term risk generally refers to known probabilities of negative outcomes, the more appropriate term in this context would be uncertainty, which refers to unknown or subjective probabilities (LeRoy and Singell, 1987; Gigerenzer, 2002). However, the terms risk and uncertainty are often used interchangeably (Breakwell, 1996; Gigerenzer, 2002). In order to make appropriate decisions in situations that involve uncertainty, but do not offer stated probabilities, people need to rely on their subjective judgments (Hertwig, 2012). These subjective judgments are referred to as risk perception throughout this article.

### The Role of Feelings When Judging Risks

When people make judgments in situations that involve risks or uncertainty, they often base their initial judgment on their perceived feelings and their intuitive reaction to the situation, which the risk-as-feelings hypothesis and the affect heuristic account for (Loewenstein et al., 2001; Slovic et al., 2004; Peters et al., 2006; Hertwig, 2012). Intuitive reactions are usually based on prior experience, but they can also be influenced by one’s current state of affect if a person lacks experience (Klein et al., 1993; Peters et al., 2006; Betsch, 2008). If someone has never experienced a similar situation before, this person might base her initial reaction on the feelings experienced during the situation at hand (Peters et al., 2006). In a risk sport setting, these feelings may include anxiety, worry or excitement, which can result in the over- or underestimation of risks (Johnson and Tversky, 1983). As a result, experienced people are assumed to rely on their affective-intuitive states (i.e., feelings based on intuition resulting from experience), while inexperienced people are assumed to rely more on affective-physical states (i.e., feelings based on the physical experience of an emotion experienced in the situation at hand) when judging risks in a sport setting. That does not imply that people do not analyze risks, but that their initial reaction is often based on intuition or affective states.

### The Role of Physical Activity When Judging Risks

Engaging in physical activity is assumed to release endorphins, which, in turn, can reduce anxiety and increase positive mood (Dishman and O’Connor, 2009). While negative mood is linked to higher risk perception, positive mood is linked to a decrease in risk perception (Johnson and Tversky, 1983). In addition, positive mood serves as an indicator for safe situations (i.e., when I feel good, it must be safe), which can result in more risk-taking behavior (Yuen and Lee, 2003).

Cognitive, affective and physical processes, such as movements of the body, influence one another, which is known as embodiment (Wilson, 2002; Wilson and Golonka, 2013). Research on embodiment has demonstrated the interplay between physical and mental processes in various contexts, but only few studies have included risk. For example, people who washed their hands after experiencing a series of gambling losses (i.e., people who washed off bad fortune) took more risks in subsequent gambles than people who had not washed their hands (Xu et al., 2012). Another study demonstrated that participants who had competed in a tennis match for 1 h showed increased risk-taking behavior in the balloon analog risk task (BART; Lejuez et al., 2003) following the match. The authors suggested that a dopamine increase that resulted from exercising encouraged reward-seeking behavior, which may entail risk. Another mechanism they suggested was fatigue, which may have led to performance errors rather than more risk-taking. However, they did not control for factors related to competition (e.g., who won or lost the game), which may have affected the results (Black et al., 2013). In a series of studies with backcountry skiers, indoor climbers and participants riding on a stationary bicycle, the perception of risks decreased during or immediately after physical activity. In these studies, physical activity was suggested to influence risk perception related and unrelated to an activity (i.e., when riding on a stationary bicycle). Furthermore, less experienced participants seemed to be more influenced by their physical activity than more experienced participants, whose judgments remained quite stable (Raue et al., 2015). In the present study, we aimed at replicating and extending these findings by also including objective measures such as participants’ climbing errors that may lead to serious injuries.

### The Present Study

As outlined above, research on embodiment suggests that being physically active influences affective states and increases positive mood. Affective states, on the other hand, are often the basis for risk perception. For example, positive mood has been shown to decrease risk perception and increase risk-taking behavior. Thus, we expected risk perception to generally decrease during the climbing activity as a result of released endorphins that increase positive affect. At the same time, we expected an increase in error rates, which may either result from a decrease in precautionary measures (due to the decrease in risk perception) or from fatigue and exhaustion. In addition to subjective risk perception, error rates are an objective measure of behavior that increase the risk for accidents.

H1: Risk perception is lower immediately after the climbing activity than before the climbing activity.
H2: Error rates increase during the climbing activity.

Experience was included as potential moderator. Indoor climbing is also accessible to inexperienced people, because it lacks natural hazards and offers permanent security through a climbing harness, which is attached to a rope. However, it is still a risk sport and may cause height anxiety, for example, resulting in nervous behavior. We assumed that more experienced climbers base their risk judgments on their intuition when being in the active state of climbing and are less affected by situational circumstances such as the climbing activity. Less experienced climbers, however, are expected to base their judgments on their current feelings, which may be triggered through being physically active. In other words, because of their lack of experience, less experienced climbers cannot rely on their intuition when judging risks, but are rather assumed to base their judgments on their current affective state (e.g., nervousness, anxiety). Since being physically active may interfere with a deliberative evaluation of the situation in addition to other affective states experienced in the situation at hand, this may make less experienced climbers more prone to making errors. As a result, the influence of the climbing activity on risk perception and error rates is expected to be stronger among less experienced climbers.

H3a: The influence of the climbing activity on risk perception is stronger among less experienced than among more experienced participants.
H3b: The influence of the climbing activity on error rates is stronger among less experienced than among more experienced participants.

## Materials and Methods

### Participants and Design

Participants were recruited in a climbing gym in a large German city. We posted advertisements in the gym and on its website. Additionally, we posted the study on the black board of the local university. We also sent out emails to the climbing gym’s email list and an adult climbing group. All participants entered a raffle for gift certificates and climbing gear.

Overall, 62 people participated in the study of which five had to be excluded because they had not filled out the questionnaire at all five time points. The remaining 57 participants (32% female) were between 18 and 57 years of age (*M* = 32.00, *SD* = 8.95), however, five of those did not indicate their age. The study consisted of climbing teams and a route could only be occupied by one team at a time. Climbing teams consisted of two participants, one person climbing and one belaying, who were approached at five points in time: (T1) the night before visiting the climbing gym, (T2) when entering the climbing gym, (T3) after the first route, (T4) before the last route, and (T5) outside the gym, after climbing. In addition, climbing errors were observed during climbers’ first and second route. All participants were familiar with climbing in general, with the climbing gym in particular, and able to judge potential risks. The gym offered numerous routes of varying levels of difficulty, but routes were equal concerning type of wall, length, steepness and safety measures. The level of difficulty was indicated at each route. Participants were free to choose the routes, but were asked to climb routes in accordance with their abilities to keep the individual challenge comparable. Since routes only differed by difficulty (i.e., size and placements of holds), but not by shape, steepness or protection measures, and since all climbers were physically able to climb the selected route, the level of difficulty should not influence objective safety.

### Materials and Procedure

We measured risk perception at five points in time using a questionnaire. The day before going to the climbing gym, participants received an online version of the questionnaire; on the day of climbing they received paper-and-pencil versions of the questionnaire. At each point in time, except for the first one, participants were asked to judge the probability of being affected by nine different climbing risks on that same day (fall, getting injured, fall due to failure of hold, failure of material, injury by falling, injury due to outside forces, injury due to others, injury due to belay errors, currently experienced level of risk) on an analog scale from 0 (*very unlikely*) to 100 (*very likely*). At the first point in time, participants were asked to judge the probability of being affected by the named climbing risks on their upcoming climbing activity the next day.

While climbing in the gym, participants were observed by two independent raters, who were also experienced climbers, during their first and their second route. The raters were instructed in using the scale, on which they recorded climbing and belaying errors. The observation was based on assessment sheets developed by the German alpine club (DAV), which categorizes climbing errors into A (error leads to immediate fall), B, C, D and E (error without severe consequences)^1^ (Funk et al., 2013). The intraclass correlational coefficients (ICC) for each error category and each route were between. 49 and 1.00 (ICC of type E errors on route 2 was 0.49, but > 0.79 in all other categories). A mean of errors was calculated over both raters. Raters were thoroughly instructed to intervene in case of the occurrence of errors in order to prevent harm to participants.

## Results

### Experience

The preferred level of difficulty when lead climbing^2^ varied among participants between grades 4 and 8 (according to the International Climbing and Mountaineering Federation, UIAA)^3^, however, grades 6 and 7 were most preferred. Furthermore, participants were asked about their climbing experience, climbing frequency and professional trainings. Years of climbing experience ranged from 1 to 45 years (*M* = 7.46, *SD* = 7.88). The majority of participants (93%) indicated that they go climbing once or several times a week. Since this was the upper end of the scale, we did not include climbing frequency in the analysis. Of all participants, 46% indicated being professionally trained (see Supplementary Table S1 for raw data).

### Risk Perception

**FIGURE 1:**
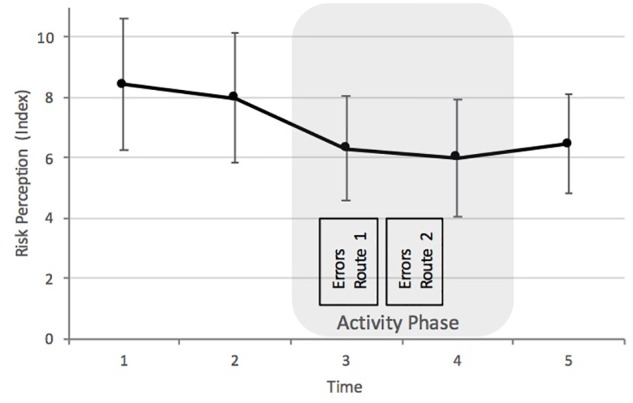
Perception of merged risks (risk index; risk of falling excluded). Error bars indicate 95% confidence intervals. Points in time are the night before visiting the climbing gym (T1), when entering the climbing gym (T2), after the first route (T3), before the last route (T4), and outside the gym, after climbing (T5). The figure also indicates the time of the error measurement.

Based on an analysis of internal consistency, we merged the climbing risks into a single index variable (risk of falling was excluded and is reported separately due to reliability reasons), all α > 0.87. We investigated how participants’ risk perception changed during the climbing activity by conducting a linear mixed model for the repeated measures of the risk perception index for each subject. We measured fixed effects of time and experience and the interactions between the two, which resulted in a total of 16 parameters. However, no significant effects were found when controlling for experience. After excluding experience, we reran the analysis using a repeated measure ANOVA and found a significant main effect of the repeated measurement on the risk perception index, *F* (4, 224) = 7.19, *p* = 0.001, η^2^_p_ = 0.11 (observed power was 0.96). The perceived likelihood of climbing risks dropped continuously until the end of the activity phase (see Figure 1). This change reached statistical significance between T1 (*M* = 8.92, *SD* = 8.19) and T3 (*M* = 6.31, *SD* = 6.51), *p* = 0.010, *d* = 0.43, as well as T1 and T4 (*M* = 5.99, *SD* = 7.28), *p* = 0.011, *d* = 0.43, between T2 (*M* = 7.98, *SD* = 8.09) and T3, *p* = 0.007, *d* = 0.48, as well as T2 and T4, *p* = 0.019, *d* = 0.42 (Bonferroni *post hoc* tests) (T5: *M* = 6.45, *SD* = 6.15; see Table 1 for results on single risks).

**Table 1:** Means, standard deviations, and results of a repeated measure ANOVA of each risk and the risk index (risk of falling excluded) at each point in time (*N* = 57).

	T1		T2		T3		T4		T5		Repeated Measure ANOVA		
	*M*	*SD*	*M*	*SD*	*M*	*SD*	*M*	*SD*	*M*	*SD*	*F*	*p*	η^2^_p_
*Risk Index*	8.92	8.19	7.98	8.09	6.31	6.51	5.99	7.28	6.45	6.15	**7.19^∗^**	0.001	0.114
*Falling*	31.00	25.57	27.72	26.26	15.53	16.26	25.32	28.02	28.37	27.92	**5.48^∗^**	0.002	0.089
Injury	12.39	14.91	8.93	10.58	6.56	8.12	7.42	8.211	9.98	7.51	**5.33^∗^**	0.003	0.087
Failure of hold	10.33	11.75	7.96	11.01	7.82	10.45	6.68	11.28	7.51	3.89	**3.17^∗^**	0.035	0.054
Material failure	6.32	10.70	5.77	10.27	5.02	9.20	4.61	8.83	3.89	8.07	**3.26^∗^**	0.039	0.055
Injury by falling	7.84	8.79	9.09	11.11	6.30	6.83	6.35	8.80	8.07	6.65	2.31	0.078	0.040
Injury due to outside forces	9.51	10.18	8.51	10.61	6.44	9.68	5.37	9.27	6.65	5.04	**4.72^∗^**	0.010	0.078
Injury through others	7.30	7.43	7.47	9.96	5.30	7.45	5.25	9.17	5.04	3.47	2.78	0.051	0.047
Injury due to belaying errors	8.05	8.30	5.86	7.56	4.88	7.28	3.63	4.00	3.47	9.98	**10.21^∗^**	< 0.001	0.154
Global risk	9.75	11.01	10.29	16.78	8.05	13.60	8.70	12.79	7.04	12.24	1.19	0.312	0.021


*^∗^significant at the 0.05 level (2-tailed).*

### Risk of Falling

**FIGURE 2:**
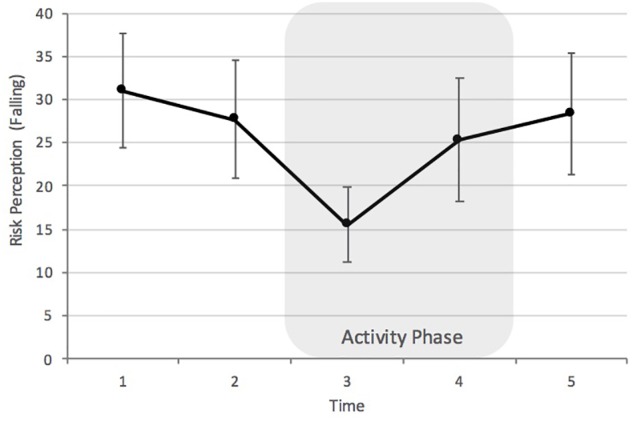
Perception of the risk of falling. Error bars indicate 95% confidence intervals. Points in time are the night before visiting the climbing gym (T1), when entering the climbing gym (T2), after the first route (T3), before the last route (T4), and outside the gym, after climbing (T5).

The risk of falling was analyzed separately using a linear mixed model. We measured fixed effects of time and experience and the interactions between the two, which resulted in a total of 16 parameters. Since there was no interaction of time and experience, *F* = 1.68, *p* = 0.328, we removed the interaction term and reran the model. We found a main effect of time, *F* (4, 106.67) = 5.58, *p* < 0.001 (see Figure 2) and a main effect of experience, *F* (1, 228.23) = 15.62, *p* < 0.001. Risk perception decreased from the inactivity phases (T1, T2) until the end of the first climbing route (T3, activity phase). This decrease reached statistical significance between T1 (*M* = 31.00, *SD* = 25.57) and T3 (*M* = 15.53, *SD* = 16.26), *p* = 0.002, *d* = 0.49, and between T2 (*M* = 27.72, *SD* = 26.26) and T3, *p* = .035, *d* = 0.43. Risk perception then significantly increased again from T3 until leaving the climbing hall at T5 (*M* = 28.37, *SD* = 27.92), *p* = 0.026, *d* = 0.38 (Bonferroni *post hoc* tests) (T4: *M* = 25.32, *SD* = 28.02; see Figure 2). A linear regression analysis with the perceived risk of falling averaged across time (*M* = 25.59, *SD* = 18.36) as dependent variable and experience as predictor indicated that more experience is associated with lower judgments of the risk of falling, *b* = -0.81, S.E. = 0.30, β = -0.35, *p* = 0.008. The correlation analysis of single risks (see Table 2) further indicates that experience may play a more significant role before the activity and toward the end or after the activity, respectively.

**Table 2:** Correlations of the risk perception variables and experience at each point in time.

	T1		T2		T3		T4		T5	
	Index	Falling	Index	Falling	Index	Falling	Index	Falling	Index	Falling
Experience	-0.097	-0.258^∗^	-0.047	-0.157	-0.052	-0.139	-0.079	-0.308^∗^	-0.146	-0.339^∗^


*^∗^significant at the 0.05 level (2-tailed).*

### Climbing Errors

Additionally, we looked at climbing and belaying errors that participants made. There were only few type B errors (*M* = 0.79, *SD* = 2.91) and only one type A error (the rope was not installed correctly into the belaying tool, which was noticed immediately by both climbers and adjusted accordingly before they started to climb), which were more severe errors. Type D errors were the most frequent (*M* = 4.11, *SD* = 3.11), followed by type C errors (*M* = 3.37, *SD* = 3.78) and type E errors (*M* = 1.86, *SD* = 1.72). The most common errors were “biting into the rope to clip it” (98 observations) and “not concentrating when belaying” (i.e., talking to a third person, not paying attention to the climbing partner; 97.5 observations). Other common errors were “incorrect handling of rope” (62 observations), “bad belaying position” (44 observations) and “left too much drag rope” (43 observations). All clips were used correctly, harnesses were closed correctly and knots were tied correctly. For the analyses error categories were combined and mean error rates were calculated.

The amount of errors increased from the first (*M* = 4.54, *SD* = 3.77) to the second climbing route (*M* = 5.62, *SD* = 4.16), *t* (56) = 2.30, *p* = 0.026, *d* = 0.27. Split by error categories, this increase in errors was statistically significant for type C errors only (*M*_1_ = 1.17, *SD*_1_ = 1.64; *M*_2_ = 2.21, *SD*_2_ = 2.54), *t* (56) = 3.98, *p* < 0.001, *d* = 0.49. We analyzed how experience influenced the amount of errors by using a linear mixed model with mean error rates as repeated measure, which resulted in a total of 7 parameters. We neither found a main effect of time, *F* = 1.13, *p* = 0.291, nor an interaction of time and experience, *F* < 1, *p* = .991, and excluded these terms from the analysis. We reran the analysis with experience as a fixed factor and found that more experienced climbers made more errors, *F* (1, 108.89) = 4.56, *p* = 0.035. We also ran a linear mixed model for each type of error and found a main effect of experience for type D errors only, *F* (1, 111.82) = 11.91, *p* = 0.001.

## Discussion

This research aimed at highlighting shifts in risk perception while engaging in risk-related leisure activities at the example of a climbing activity. In this study, we examined the influence of the climbing activity and climbing experience on risk perception and error rates during indoor climbing. We found that participants’ perception of climbing risks generally decreased during the activity phase as opposed to the pre-activity phase, which supports Hypothesis 1. Second, participants’ climbing errors generally increased from the first to the second measurement of errors, which supports Hypothesis 2. While the amount of errors was higher for more experienced participants, we did not find support for Hypothesis 3b that the climbing activity influences error rates among less experienced climbers. Third, while we did not find significant interactions of experience and the repeated measures of risk perception, a correlation analysis indicated a significant relationship of experience and the perceived risk of falling before and at the end of the activity. This may offer some support for Hypothesis 3a, potentially indicating a stronger decrease in the perceived risk of falling during the activity phase among less experienced participants as opposed to more experienced participants.

In sum, our findings are consistent with our previous research indicating that physical activity can decrease risk perception (Raue et al., 2015), but further studies investigating this relation are necessary. This finding is alarming, especially in higher risk sports (including those that do not offer a protection), where important risk judgments are constantly made while being physically active. A climbing gym is a rather safe environment, but serious accidents still happen. According to the annual accident reports of the German Alpine Club (Deutscher Alpenverein, 2012, 2016), human error is the cause of almost every single accident in climbing gyms. The attenuating effect of physical activity on risk perception could be fatal when climbing outdoors or skiing in the backcountry. Higher experience may weaken the influence of physical activity on risk perception, but more research is needed to understand whether there is a relationship. Error rates, however, increased with more experience.

The general increase in errors among more experienced climbers was an unexpected finding. One possible explanation is that these climbers were more exhausted due to climbing more challenging routes and were therefore more prone to making errors. Although we asked the climbers to choose routes in accordance with their abilities, we cannot rule out that varying levels of ability and route difficulty led to varying levels of exhaustion among climbers. For instance, a study on mountaineering accidents revealed that fatigue is among the most common reasons for accidents of downhill skiers (Chamarro and Fernández-Castro, 2009). However, as we did not measure physical exhaustion and error measurement took place in the first two routes of the day, this explanation remains speculative. The climbers could also just have felt more comfortable in their environment after having climbed their first route, which may have increased their confidence. Due to their experience and intuitive knowledge, more experienced climbers who feel confident in their skills may soften standard rules without putting themselves on the brink of severe risks. While this explanation also comes with some speculation, the influence of experience on minor type D errors specifically may speak in favor for this line of reasoning.

The lack of physical measures (e.g., heart rate, lactate concentration) to capture exhaustion and fatigue poses some limitations to our conclusion. Further, a possible confound of physical activity with the outcome of that physical activity (i.e., climbing) cannot be ruled out. As a result, the constant experience of climbing without negative consequences such as falling may have decreased risk perception. However, earlier studies we conducted showed that also risks that were unrelated to the physical activity were judged as lower during the activity phase (see Raue et al., 2015, Study 3). Future studies on the relationship of physical activity and risk perception should consider these potential confounds, measure risk perception during the physical activity and also include physiological measures. Through measures that capture levels of exhaustion, but also hormone levels (e.g., endorphin), we could get an idea of underlying mechanisms. We assume that an increase in endorphin levels through physical activity increases positive mood, which leads to reduced risk perception and more risk taking. Endorphin levels could be measured by taking blood samples from participants. Also, increased dopamine levels could be an underlying mechanism as suggested by Black et al. (2013). However, short-term variations of dopamine levels might be difficult to measure in a field study. We also assume that less experienced climbers may be more affected by changes in affective states during physical activity since they base judgments on their current affective state rather than intuition (as it is the assumed for experts). However, it is challenging to measure potential mediators without significantly changing the situation at hand. Therefore, responses to these measures need to be short and effortless. One idea for future studies is to measure unspecific mediators like negative mood or risk perception with one-item scales (cf. Houtman and Bakker, 1989) or two-dimensional grids like the affect grid, which captures the dimensions pleasure-displeasure and arousal-sleepiness (Russell et al., 1989). Thus, future studies should especially focus on these potential underlying mechanisms to get a better understanding of how bodily states influence risk perception.

Especially in higher risk sports people have to make important decisions while being active. The growing popularity of these activities comes with an increasing number of inexperienced participants. Experience may weaken the influence of physical activity on the perception of risks. However, based on our results we cannot make inferences concerning the accuracy of climbers’ risk perception. It is assumed that more experience is linked to more accurate risk perception. Higher risk perception was associated with less experience in this study, suggesting less accuracy with less experience.

## Conclusion

In conclusion, people who engage in risk-related leisure activities need to be aware that being physically active could influence their risk perception and should pause to concentrate on the task at hand. This may especially be the case for those with less experience. Nonetheless, future research needs to investigate whether physical activity distorts or promotes accuracy and whether inactivity leads to an over- or underestimation of risks. People also need to be aware that their error rate may increase during physical activity. If exhaustion is the reason for this increase, they need to listen to their bodies and take more breaks during the activity in order to avoid accidents. However, future research needs to further investigate how activity affects risk perceptions, and how people can be trained to make accurate risk judgements and decisions while being active in order to keep popular risk-related leisure activities as safe as possible.

## Ethics Statement

Our study was conducted in full accordance with the ethical guidelines of the German Association of Psychologists (DGPs) and the American Psychological Association (APA). Following the ethical guidelines of the Department of Psychology at Ludwig Maximilian University (LMU), no substantive assessment of the Ethics Committee was required for this research project. By the time the data were acquired, it was not customary at LMU Munich, nor at most other German universities, to seek ethics approval for studies that involve no sensitive personal data, affect no vulnerable groups and pose no risks to participants. Therefore, ethical approval was not required for this study in accordance with the national and institutional guidelines. The study exclusively used of questionnaires. No identifying information was obtained from participants. Every participant was fully informed about the study and gave informed consent by agreeing to participate. Participants were explicitly informed that all data are treated confidentially and that they may withdraw from the study at any time without giving explanations.

## Author Contributions

MR drafted the manuscript, conducted the analysis, and interpreted the data. RK acquired the data and supported the data analysis and interpretation. EL and BS contributed substantially to the interpretation of the data. RK, EL, and BS revised the draft critically. All authors finally approved the version to be published and agreed to be accountable for all aspects of the work in ensuring that questions related to the accuracy or integrity of any part of the work are appropriately investigated and resolved and contributed to the conception of the work.

## Conflict of Interest Statement

The authors declare that the research was conducted in the absence of any commercial or financial relationships that could be construed as a potential conflict of interest.

## Footnotes

1 Type A errors lead to a fall, type B errors lead to a fall in combination with one other event, type C errors lead at least to an injury in combination with another event or to a fall in combination with several other events, type D errors could lead to an injury, type E errors usually do not have any consequences.

2 Lead climbing is defined as ascending a route while periodically placing protection for safety in the event of a fall. In contrast, top-rope climbing requires a pre-placed anchor at the top of the route.

3 Grades of climbing routes vary among countries, but in Germany the standard of the UIAA is used, which is an open-ended scale. More precise difficulties are indicated with – (at the lower end) and + (at the higher end). The hardest climbing route is currently indicated with grade 12-.
